# Genome-Wide Transcriptomic and Metabolomic Analyses Unveiling the Defence Mechanisms of *Populus tremula* against Sucking and Chewing Insect Herbivores

**DOI:** 10.3390/ijms25116124

**Published:** 2024-06-01

**Authors:** Filip Pastierovič, Kanakachari Mogilicherla, Jaromír Hradecký, Alina Kalyniukova, Ondřej Dvořák, Amit Roy, Ivana Tomášková

**Affiliations:** 1 Faculty of Forestry and Wood Sciences, Czech University of Life Sciences Prague, Kamýcká 129, CZ 165 00 Praha, Suchdol, Czech Republic; pastierovic@fld.czu.cz (F.P.); mogilicherla@fld.czu.cz (K.M.); hradecky@fld.czu.cz (J.H.); diuzheva@fld.czu.cz (A.K.); dvorak18@fld.czu.cz (O.D.); roy@fld.czu.cz (A.R.); 2 ICAR-Indian Institute of Rice Research (IIRR), Rajendra Nagar, Hyderabad 500030, Telangana, India

**Keywords:** *Populus tremula*, aphids, spongy moth, transcriptome and metabolomics, reference gene analysis, RT-qPCR, induced defence

## Abstract

Plants and insects coevolved as an evolutionarily successful and enduring association. The molecular arms race led to evolutionary novelties regarding unique mechanisms of defence and detoxification in plants and insects. While insects adopt mechanisms to conquer host defence, trees develop well-orchestrated and species-specific defence strategies against insect herbivory. However, current knowledge on the molecular underpinnings of fine-tuned tree defence responses against different herbivore insects is still restricted. In the current study, using a multi-omics approach, we unveiled the defence response of *Populus tremula* against aphids (*Chaitophorus populialbae*) and spongy moths (*Lymantria dispar*) herbivory. Comparative differential gene expression (DGE) analyses revealed that around 272 and 1203 transcripts were differentially regulated in *P. tremula* after moth and aphid herbivory compared to uninfested controls. Interestingly, 5716 transcripts were differentially regulated in *P. tremula* between aphids and moth infestation. Further investigation showed that defence-related stress hormones and their lipid precursors, transcription factors, and signalling molecules were over-expressed, whereas the growth-related counterparts were suppressed in *P. tremula* after aphid and moth herbivory. Metabolomics analysis documented that around 37% of all significantly abundant metabolites were associated with biochemical pathways related to tree growth and defence. However, the metabolic profiles of aphid and moth-fed trees were quite distinct, indicating species-specific response optimization. After identifying the suitable reference genes in *P. tremula*, the omics data were further validated using RT-qPCR. Nevertheless, our findings documented species-specific fine-tuning of the defence response of *P. tremula,* showing conservation on resource allocation for defence overgrowth under aphid and moth herbivory. Such findings can be exploited to enhance our current understanding of molecular orchestration of tree responses against herbivory and aid in developing insect pest resistance *P. tremula* varieties.

## 1. Introduction

Over approximately 350 million years, plants and insects have coevolved, resulting in a spectrum of beneficial and detrimental interactions between the two groups [1,2]. Beneficial interactions encompass insect-mediated processes such as pollination and seed dispersion, providing mutual advantages to both interaction partners. Conversely, negative interactions involve insect predation, frequently damaging the host [1,2]. To repel insect pests and pathogens, plants have evolved a variety of morphological and biochemical defences, such as constitutive and induced defence, that are highly effective and dynamic [3,4,5,6,7,8,9,10]. Because plant development and defence are mutually exclusive, induced defence is more frequently triggered in response to herbivore attacks than constitutive defence to optimally utilize the energy budget [4,5,10,11]. After plants perceive the herbivory, damage-associated molecular patterns or herbivore-associated molecular patterns trigger the herbivore-inducible defences.

The repercussions of global climate change extend across all layers of ecosystems, predominantly manifesting in adverse impacts on forest stands worldwide. Over 50% of tree damage worldwide is attributed to biotic causes, with insect herbivores being one of the principal stressors [12]. It corresponded that the negative alterations favour insects, even though the intricate interactions between insect herbivores and host trees are unpredictable [13]. Drought and higher temperatures weaken the tree defence, encouraging insect abundance and dispersal across larger geographic areas while reducing the duration of their generation [14,15].

Interestingly, trees are constantly under pressure to resist multiple herbivores of the same or different feeding guild. Current research indicates that trees can tailor defences against different insect herbivores and their sequential attacks using a plastic defence strategy [16]. However, understanding the tree defence tailoring for two herbivores from different feeding guilds (i.e., sucking and chewing) is very limited. Next-generation sequencing (RNA-Seq) and metabolomics have emerged as valuable tools for unveiling plant signalling networks in response to herbivory [17,18]. Hence, the current study attempted to understand how *P. tremula* responds to herbivory by insects from different feeding guilds. To delve into this, we employed a combination of transcriptomics and metabolomics on *Populus* leaves, comparing those with and without sucking and chewing insect infestation. By combining transcriptomic and metabolomic data, we quantitatively map transcripts to specific metabolic pathways involved in resistance against both types of insect feeding. It has been found that insect infestation has led to notable alterations in tree primary metabolism, affecting photosynthesis, carbohydrate and amino acid pathways, and secondary metabolites related to flavonoids. Nevertheless, these findings deepen our understanding of different herbivore-induced plant defences in *P. tremula*, providing insights for developing strategies against pests like aphids and spongy moths.

## 2. Results

### 2.1. Metabolomic Analysis

**Figure 1:**
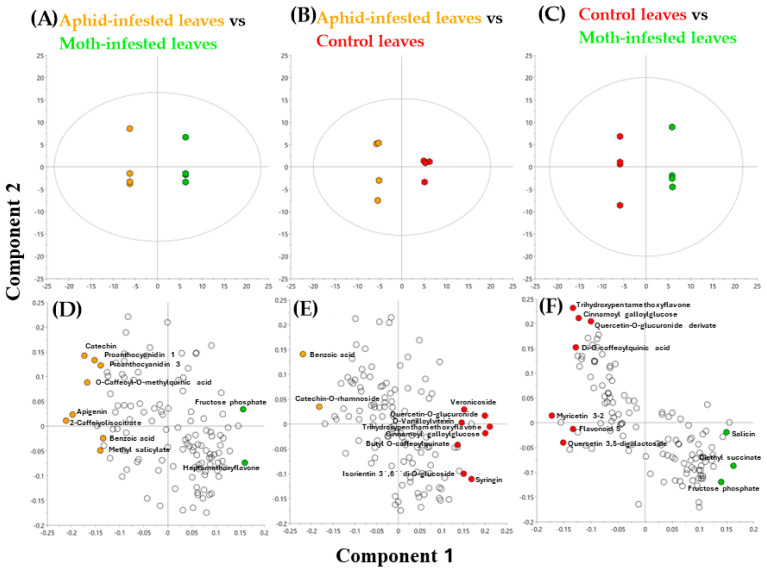
Xy OPLS-DA score plots for two groups models: (**A**) aphid-infested leaves vs. moth-infested leaves, (**B**) aphid-infested leaves vs. control leaves, and (**C**) control leaves vs. moth-infested leaves. Hotteling ellipse 95%. (**D**–**F**) OPLS-DA loading plots for models (**A**–**C**) with the ten most important compounds for separation highlighted, selected from variable importance plot. The colours of highlighted compounds correspond to sample classes in respective models.

**Figure 2:**
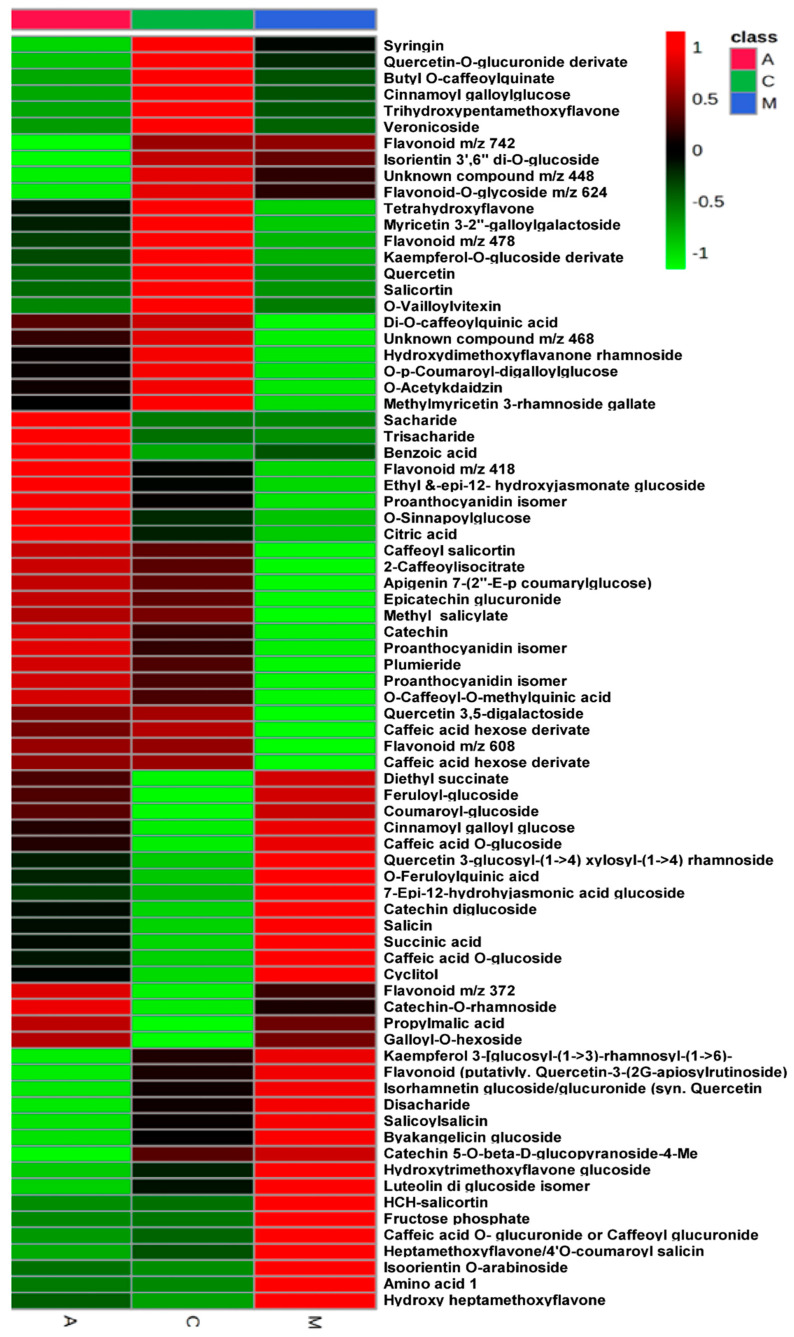
Heatmap for differentially abundant metabolites (DAMs) in *Poplar* samples tested in the current study. A—Aphid-infested treatment, C—control, M—Moth-infested treatment.

Initial principal component analysis (PCA) revealed a tendency for clustering (explained variance by the first two principal components was 76%) (Figure 1A–C). Identified metabolites in the sucking and chewing insect treatment groups differed significantly from the control group, as demonstrated by PCA of the differentially accumulated metabolites. Following Orthogonal Partial Least Squares Discriminant Analysis (OPLS-DA) showed clear separation (R^2^X(cum) = 72%, R^2^Y(cum) = 85%, Q^2^(cum) = 56%) (Figure 1). For two-group models, in the case of aphid vs. control comparison, the resulting OPLS-DA parameters were R^2^X(cum) = 65%, R^2^Y(cum) = 99%, Q^2^(cum) = 75%), for moth vs. control R^2^X(cum) = 98%, R^2^Y(cum) = 100%, Q^2^(cum) = 98%) and for aphid vs. moth R^2^X(cum) = 97%, R^2^Y(cum) = 100%, Q^2^(cum) = 99%). Despite the relatively low number of samples measured from the created models, it can be concluded that damage by the selected pests strongly affected the non-volatile metabolome of tested leaves. Lower parameters for the model considering control vs. aphid-infested samples result from larger variability in between samples of aphid-infested leaves, also resulting in lower predictive power (Q_2_(cum); based on multiple internal model cross-validation). A total of 78 differentially regulated metabolites were found across different treatments and comparisons (VIP > 1 and log2FC ≥ 1) (Figure 1 and Figure 2). We discovered that among the differential abundance metabolites (DAMs), fructose phosphate and benzoic acid were the most significantly enriched; we also displayed the top 10 metabolites in individual treatments compared to each other (Figure 1D–F and Figure 2, Table 1).

### 2.2. DGE Analysis on Poplar Leaves Infested by Sucking and Chewing Pests

**Figure 3:**
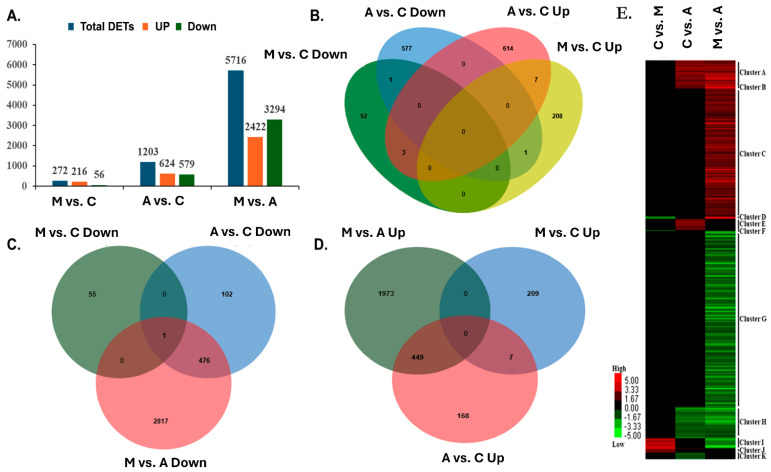
Differential gene expression and cluster analysis (treatment legend: A—aphid-infested treatment, C—control, M—moth-infested treatment). (**A**) Number of genes differentially regulated in various comparisons. (**B**) Venn diagram showing comparisons between different treatments. (**C**) Venn diagram showing comparisons between all down-regulated genes. (**D**) The Venn diagram shows comparisons between all upregulated genes. (**E**) Hierarchical cluster analysis of DEGs in three comparisons. The letters (A–K) indicate major groups identified by cluster analysis. Red colour indicates upregulation (>2.0 fold), green colour indicates downregulation (<−2.0 fold), and black indicates no change as compared to respective controls.

We investigated the gene expression profiles of *P. tremula* leaves infected by aphids and moths and their corresponding control samples. We analysed the differentially expressed genes in leaf samples attacked by sucking and chewing insects and compared them with their respective control groups. Five biological replicates were employed for each treatment, yielding fifteen samples. We obtained 13.5 Gb of data, with each sample generating more than 12.5 Gb of clean data and a Q30 base percentage exceeding 80% (Supplementary File S1). A total of 7191 differentially expressed genes (DEGs) were found following treatment with sucking and chewing insect attacks; off these, 216 and 56 were up- and downregulated in spongy moth infestation and whereas 624 and 579 were up- and downregulated when compared to respective control leaf samples, respectively. Between the aphid and spongy moth comparison, 2422 and 3294 genes were differentially regulated (*p*-adjust value < 0.05 and log2FC > 1) (Figure 3A). Furthermore, hierarchical clustering was performed to illustrate the expression pattern of DEGs. Poplar response against aphids and spongy moths feeding revealed significant differences, suggesting that the poplar gene expression level varies depending on the insect feeding style (Figure 3E). The Venn diagrams also endorse the unique plant response to different insect feeding as different genes were up or down-regulated after moth and aphid feeding (Figure 3B–D).

**Figure 4:**
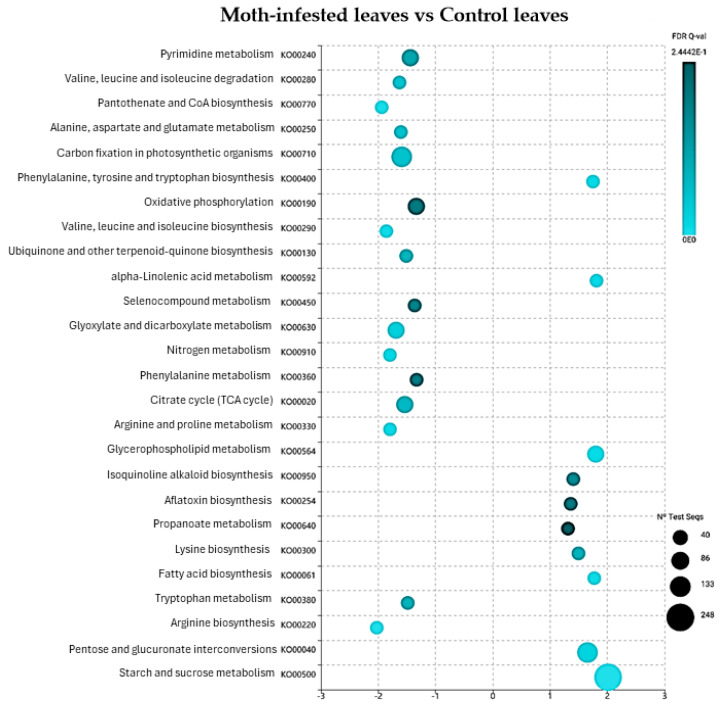
Pathway enrichment in the treatment of moth-infested leaves compared to control leaves according to the number of identified DEGs for individual metabolic pathways.

**Figure 5:**
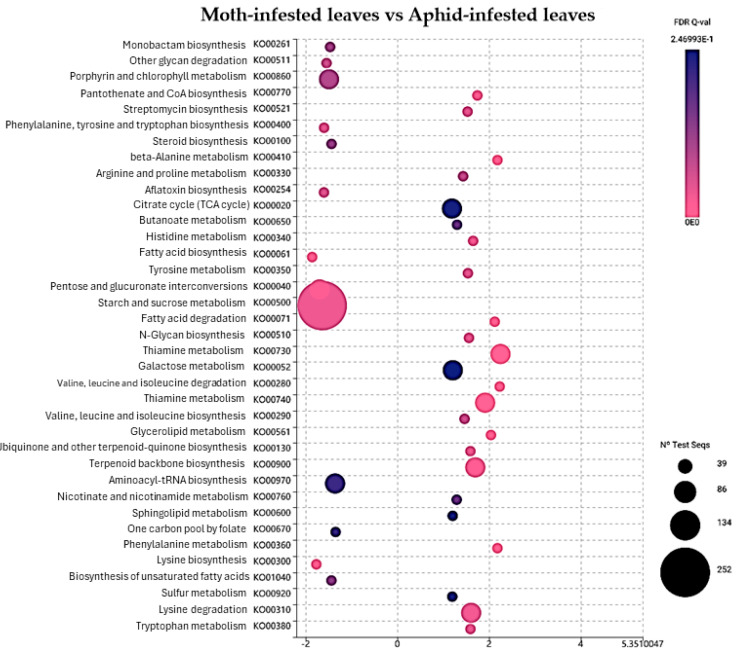
Pathway enrichment in the treatment of moth-infested leaves compared to aphid-infested leaves according to the number of identified DEGs for individual metabolic pathways.

**Figure 6:**
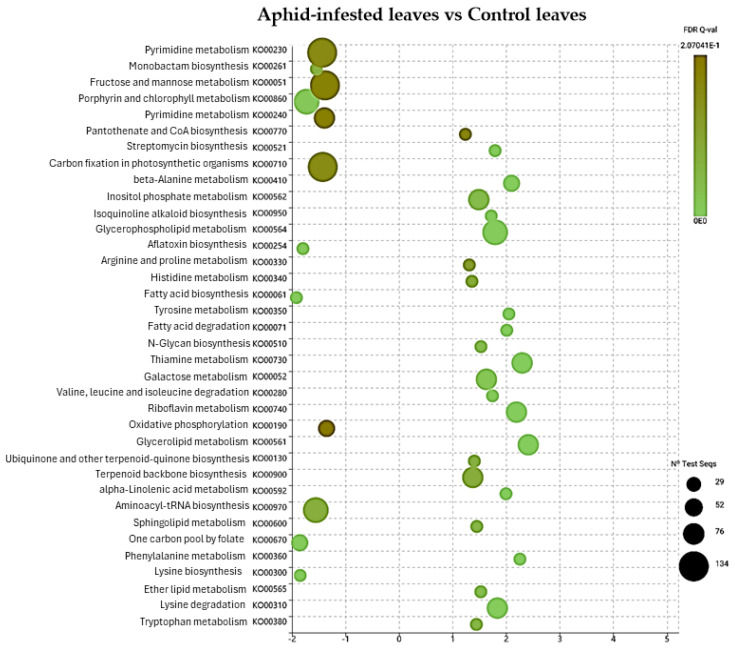
Pathway enrichment in the treatment of aphid-infested leaves compared to control leaves according to the number of identified DEGs for individual metabolic pathways.

**Figure 7:**
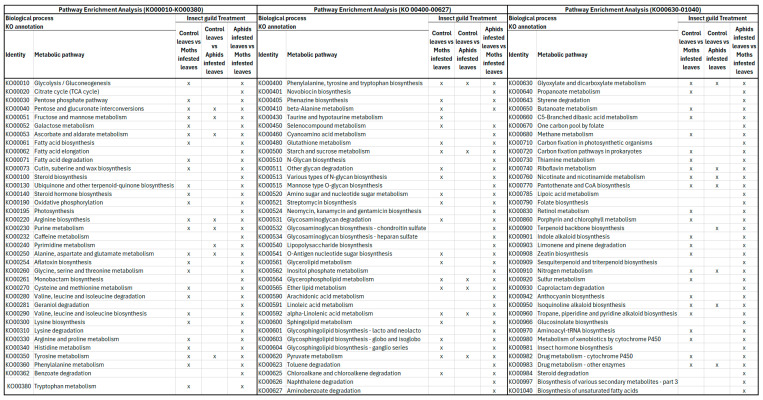
Pathway enrichment analysis. The “X” means that a DEG has been identified in the individual metabolic pathway.

Further examination revealed that identified DEGs distributed across comparisons of distinct KEGG pathways (Figure 4, Figure 5, Figure 6 and Figure 7). Specifically, DEGs were associated with pathways involved in genetic information processing, encompassing processes such as folding, sorting, degradation, transcription, and translation. Additionally, DEGs were linked to various metabolic pathways, including carbohydrate metabolism, lipid metabolism, amino acid metabolism, and biosynthesis of secondary metabolites (Figure 7). Furthermore, DEGs were implicated in cellular processes, environmental adaptation, and signal transduction pathways. In spongy moths, compared to the control group, there was an upregulation in genes associated with oxidoreductase activity, DNA binding, transcription regulatory activity, and DNA-binding transcription factor activity. At the same time, starch and sucrose metabolism increased, and carbon fixation in photosynthetic metabolism decreased (Figure 4). Similarly, when compared to spongy moths with aphids, thiamine metabolism had an upregulation and a significant downregulation in starch and sucrose metabolism (Figure 5). In aphids, compared to the control group, genes related to transferase activity, glycosyl transferase activity, lipid metabolic process, and hydrolase activity were upregulated, while Glycerophospholipid metabolism increased, and arginine and proline metabolism, fructose and mannose metabolism, and carbon fixation in photosynthetic organism metabolism were notably downregulated (Figure 6). Complete visualization of PEA (pathway enrichment analysis) in all treatments is shown in Figure 7.

### 2.3. Reference Gene Selection

**Figure 8:**
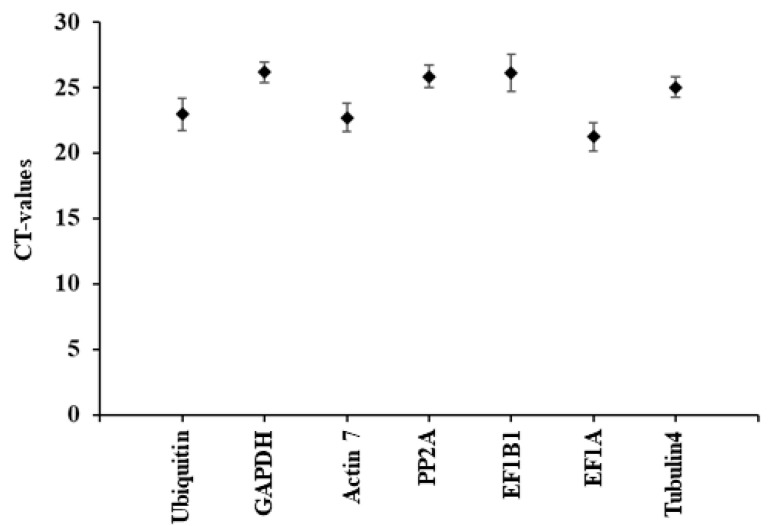
Identification of reliable housekeeping genes. Seven housekeeping genes’ Ct values in control, aphid- and moth-infected *Populus* leaf samples varied. Total RNA was obtained and converted to cDNA to calculate Ct values. The cDNA and gene-specific primers were then utilized in RT-qPCR. Ct values are displayed as the mean ± SE.

Using a standard curve created with StepOne^TM^ Software v2.3 and a known concentration of cDNA template, seven reference genes were filtered out based on their PCR amplification efficiency (Supplementary Table S1). The genes are expressed in *P. tremula*, according to the amplified product examined on the agarose gel. An expected amplicon size determined each gene’s specificity, and the amplification efficiency range was 80–120%. For these reference genes, the coefficient of determination (R2) varied from 0.91 to 1.00 (Supplementary Table S1). The single peak was shown by the amplification specificity of each gene in the RT-qPCR examined using a melt curve analysis (Supplementary Figure S3). All potential reference genes had average Cq values between 21 and 26 (Figure 8).

Based on the overall ranking by geNorm, NormFinder, BestKeeper, ∆Ct, and RefFinder, PP2A, GAPDH, and Act7 were designated as the highly stable genes across the treatments (Table 2; Supplementary Figures S2 and S3). EF1B1 and EF1A were the least stable genes as calculated by all the algorithms (Table 2; Supplementary Figures S2 and S3).

### 2.4. Gene Expression Validation

**Figure 9:**
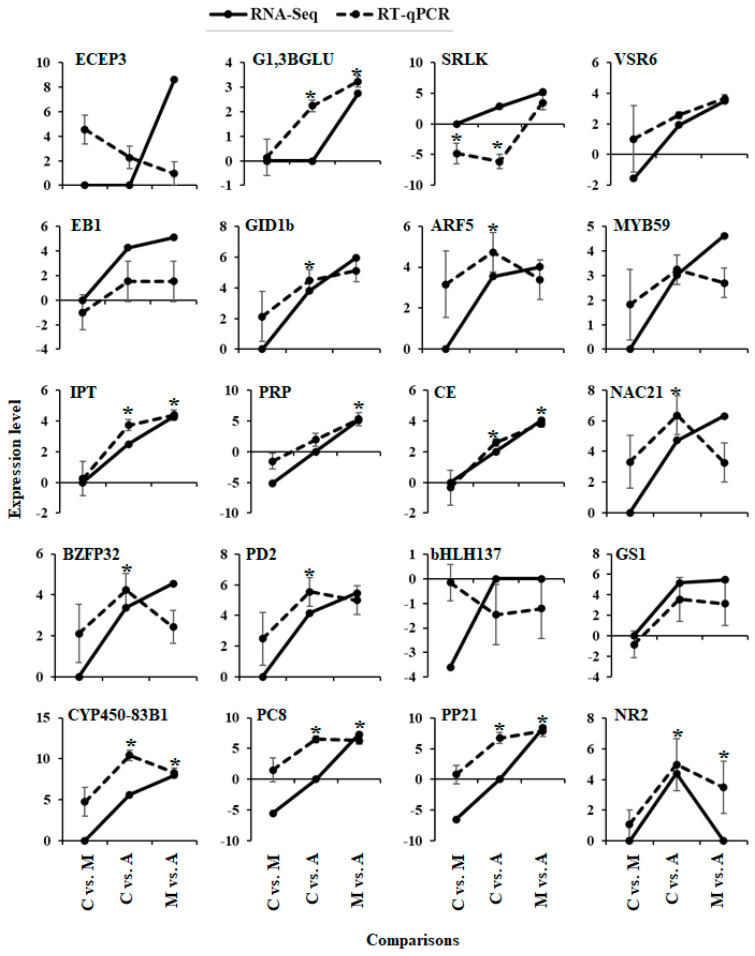
Comparison of transcriptome and RT-qPCR data for the expression of 20 genes in different feeding insects attack Poplar leaf samples (treatment legend: A—aphid-infested treatment, C—control, M—moth-infested treatment). The *x*-axis represents different comparisons of analysed samples and the *y*-axis represents the log^2^ fold change of RNA seq (*n* = 4) and RT-qPCR (*n* = 4). * represents *p* < 0.05.

To corroborate the transcriptome results, RT-qPCR was performed on twenty physiologically significant DEGs associated with defence (Figure 9). Even though certain changes in the RT-qPCR data were not statistically significant, there was sufficient agreement between the transcriptomic and RT-qPCR data regarding the expression patterns of the twenty identified DEGs. The results showed how reliable the transcriptome discoveries were made in this study.

## 3. Discussion

Poplar stands out as a key forest species due to its remarkable economic significance, rapid growth, simple vegetative reproduction, and ample genomic data, rendering it a prime candidate for the study of forest genetics, genomics, and breeding [19]. However, as a tree cultivated in open fields, poplar faces escalating environmental risks, particularly heightened biotic stresses, including insect attacks exacerbated by global warming [20]. However, *Populus* trees in southeast Asia, northeast Africa, Europe, and the East and West American continent suffer harm from over 100 insect species that belong to different insect groups, including Lepidoptera, Hemiptera, Diptera, Hymenoptera, and Coleoptera [21]. Therefore, it is critical to manage insects that harm *Populus* quantity and quality by using cultivars that are resistant to them. Plants have developed robust defence strategies against insect attacks, encompassing morphological traits, mechanistic barriers like trichomes and hairs, as well as chemical defences involving genes and pathways associated with various mechanisms [22,23,24,25]. Plant memories of previous biotic stresses can often facilitate its quick response to insect feeding [26].

The recent reports of high-quality genomes of spongy moths, aphids, *P. tremula*, and other “omics” technologies open up the scope for a higher understanding of the interactions between numerous feeding behaviour insects and plants [27,28,29,30,31,32]. Information on genetic diversity in the host response to insect infestation is needed for developing plants against pest resistance and insect control [5,33]. In this study, we examined the genes and metabolites expressed and accumulated differently by analysing the transcriptome and metabolome of leaves attacked by aphids and spongy moths compared to control leaves. We aimed to investigate the mechanisms underlying tree resistance to insects, specifically those that suck and chew on trees.

### 3.1. Response of Hormones Signalling Pathways after Aphid and Spongy Moth Infestation

**Figure 10:**
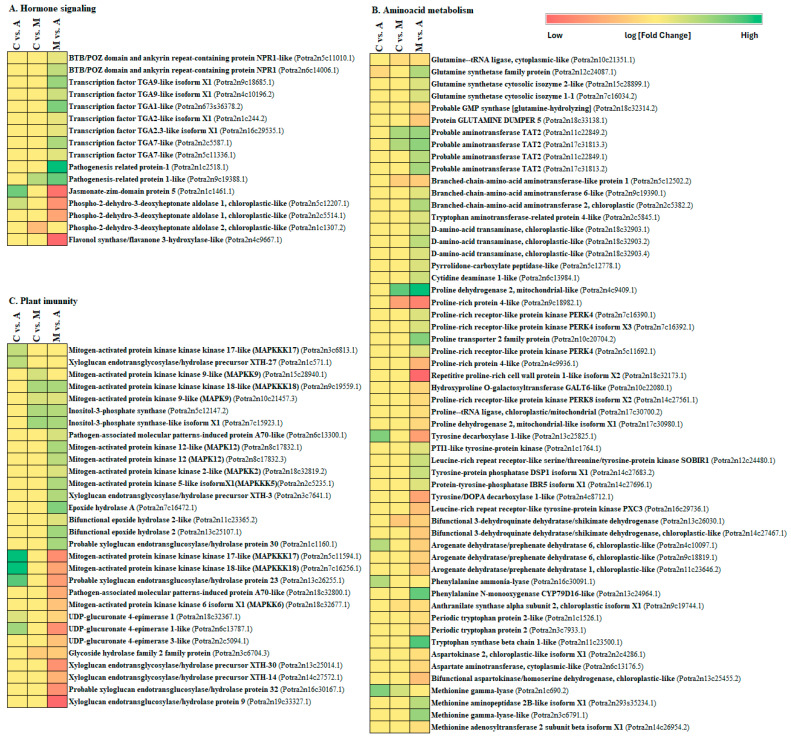
Impact of insect feeding treatment on key gene expression in *Poplar* leaves (treatment legend: A—aphid-infested treatment, C—control, M—moth-infested treatment). Genes are differentially regulated in (**A**) hormone signalling, (**B**) plant immunity, and (**C**) Amino acid metabolism pathways.

The primary signal-transduction pathways in plants that underlie induced defence against herbivorous insects are jasmonic acid (JA) and salicylic acid (SA), which often exhibit both signalling pathways that can function additively or synergistically, though they typically behave antagonistically [34,35,36]. Sap-sucking insects such as aphids and whiteflies, when they attack plants, activate genes related to SA metabolism, leading to SA accumulation in infected plants. For instance, the green peach aphid (*Myzus persicae*) feeding induces the accumulation of SA-inducible transcripts in *Arabidopsis thaliana* [37,38,39], whereas silverleaf whitefly (*Bemisia tabaci*) infestation increases SA-inducible gene transcripts in both *A. thaliana* and tomato plants [40,41]. SA has been observed to exert a detrimental effect on the growth of phloem-feeding aphids and plays a crucial role in activating plant defences against these insects [42,43,44,45]; however, salicylic acid has also been found to elicit either a neutral or even a beneficial effect on the growth of numerous other phloem-feeding insects [40,46,47,48]. In this study, in aphid infestation, we observed significant upregulation of key genes involved in salicylic acid (SA) transduction, including NPR1 (BTB/POZ domain and ankyrin repeat-containing protein NPR1, BTB/POZ domain, and ankyrin repeat-containing protein NPR1-like), PR-1 (pathogenesis-related protein-1 and pathogenesis-related protein 1-like), and TGA (transcription factor TGA1-like, transcription factor TGA2-like isoform X1, transcription factor TGA2.3-like isoform X1, transcription factor TGA7-like, and transcription factor TGA9-like isoform X1) when compared to spongy moth infestation (Figure 10A).

Plants have developed adaptive defences against chewing insects by inducing proteins, including polyphenol oxidases (PPOs) and proteinase inhibitors (Pis), which interrupt insect feeding and hinder insect growth [49,50]. Exposure of potato and tomato plants to Colorado potato beetles (*Leptinotarsa decemlineata*) prompts the activation of proteinase inhibitors (PIs), effectively inhibiting the activity of digestive proteinases within the insect’s gut [51]. Jasmonic acid (JA), induced by chewing insects and wounding, triggers the expression of defensive proteins like Pis and PPOs [49,52]. Caterpillars feeding JA-deficient tomato mutants exhibit higher survivorship and weight gain than wild-type plants [49,53]. Exogenous application of JA or methyl jasmonate (MeJA) boosts plant resistance to herbivores and stimulates defensive protein expression in tomatoes [54,55,56,57,58]. This highlights JA’s pivotal role in regulating plant defences against herbivores, with a JA-mediated pathway identified from insect attack to defensive gene expression in plants [39,47,59,60,61]. In this study, our findings align with prior research, indicating that feeding by spongy moth insects triggers the activation of the JAZ (jasmonate-zim-domain protein 5) gene, which plays a vital role in JA biosynthesis. (Figure 10A, Supplementary File S2). When plants activate a JA signalling pathway, they become resistant to phloem-feeding and chewing insects [40,62,63]. Given that SA and JA exhibit antagonistic behavior, with SA inhibiting the buildup of JA and JA-inducible gene expression [64,65,66], it is theorized that numerous phloem-feeding insects have developed a strategy to dampen or undermine JA-inducible plant defences by stimulating the SA-inducible pathway [40,46,48,67].

The phospho-2-dehydro-3-deoxyheptonate aldolase enzyme catalyses the first of seven steps of chorismite biosynthesis and the final common precursor of all three aromatic amino acids as well as PABA, ubiquinone, and menaquinone. An essential component of the flavonoid biosynthesis pathway, flavanone 3-hydroxylase (F3H) regulates the accumulation of anthocyanidins and flavonols. When the sugarcane aphid (*Melanaphis sacchari*) infected the susceptible and resistant sorghum plants, it was observed that the infected plants’ expression of flavonoid 3′-5′hydroxylase (F3′5′H) at day 10 and phorismi-2-dehydro-3-deoxyheptonate aldolase at days 10 and 15 was lower than that of the uninfested control plants [68]. In this study, genes related to chorismite biosynthesis and flavonoid biosynthesis pathways, including Phospho-2-dehydro-3-deoxyheptonate aldolase 1, Phospho-2-dehydro-3-deoxyheptonate aldolase 2, and Flavonol synthase/flavanone 3-hydroxylase-like, exhibit downregulation during aphid infestation whereas upregulation during spongy moth infestation (Figure 10A). In addition to direct feeding damage, aphids are vectors for plant diseases such as tospoviruses. Some studies suggested that viruses can manipulate plant defences by interacting with the SA and JA signalling pathways [69]. Our findings underscore the pivotal roles played by the SA and JA signalling pathways in the induction of plant defence mechanisms in response to sucking and chewing insect feeding.

### 3.2. Plant Immune Defence against Aphid and Spongy Moth Infestation

The interactions between plants and pathogens and plants and insects are known to share certain responses. Plants can fend off a pathogenic invasion and protect against insect damage and predation because of receptors on their cells that recognize pathogen-associated molecular patterns (PAMPs) and herbivore-associated molecular patterns (HAMPs) and then activate defence signalling pathways in the plant [36]. When insects attack plants, mitogen-activated protein kinases (MAPKs) get activated and regulate the plant defence induction, phytohormonal dynamics, transcription of genes relevant to defence, and synthesis of defence metabolites [70]. The MAPK cascade progresses through three sequential steps: MAPKKK phosphorylates MAPKK, which subsequently phosphorylates MAPK, and this activation of MAPK initiates a downstream cascade of events, influencing changes in plant hormone levels, restructuring the transcriptome and proteome, ultimately fortifying the plant’s defence mechanisms against insect attacks [71]. In this study, the PAPM (pathogen-associated molecular patterns-induced protein A70-like) and MAPK (MAPKKK18) associated signalling pathways were identified as differentially expressed during the sucking and chewing insects attack (Figure 10C, Supplementary File S2). However, in spongy moth infestation, MAPKKK17 and UDP-glucuronate 4-epimerase 1-like genes show upregulation, whereas in aphid infestation, MAPKK9, MAPK9, MAPK12, MAPKK2, MAPKKK5, Inositol-3-phosphate synthase, Epoxide hydrolase A, and Bifunctional epoxide hydrolase 2 genes exhibit upregulation (Figure 10C). These genes exhibit activities linked to various plant defence mechanisms, including programmed cell death, maintenance of homeostasis, accumulation of reactive oxygen species, hypersensitive response, cell wall reinforcement, and induction of defence-related genes through stomatal closure. These findings underscore the significance of DEGs related to MAPK signalling and plant–pathogen interactions in facilitating plant-induced defence against both sucking and chewing insect attacks, aligning with prior research findings and corroborating existing literature [5,72].

### 3.3. Primary Metabolism Alteration after Aphid and Spongy Moth Infestation

Insect attacks trigger a range of alterations in plant primary metabolism, including carbohydrate and nitrogen processes, as well as the composition and levels of amino acids that influence a plant’s ability to withstand insect infestations [73,74]. Many plants synthesize defensive compounds from amino acid precursors like secondary metabolites and glucosinolates because amino acids are a primary nitrogen source, and their abundance in sap is a critical determinant of insect survival [75,76,77]. For instance, in *Arabidopsis*, caterpillar feeding activated the genes involved in amino acid biosynthesis and sulfur assimilation, which are critical for cysteine and methionine production and can lead to the accumulation of defence-related compounds like glucosinolates, mainly derived from methionine and tryptophan [74,78]. Similarly, tomato plants respond to foliar herbivory by accumulating tryptophan in systemic tissues, potentially fueling the production of defensive molecules [79]. Thus, herbivore-induced amino acid biosynthesis likely facilitates the synthesis of defence compounds in certain plant defence scenarios. Insects that feed on plants often trigger changes in gene expression within the plants, leading to alterations in amino acid metabolism. This is particularly noticeable with aphids, where substantial evidence indicates that their feeding directly increases the levels of free amino acids. For instance, the green bugs (*Schizaphis graminum*) known to cause chlorosis in wheat plants have been shown to boost the essential amino acid content in the plant’s phloem sap [80]. Similarly, Japanese rowan (*Sorbus commixta*) leaves infested with apple-grass aphids (*Rhopalosiphum insertum*) exhibit a significant increase in amino acid excretion compared to unaffected leaves [81]. However, the precise mechanisms behind these observations are not fully understood, and it is likely that these herbivores enhance their diet’s amino acid content through a combination of mechanisms such as increased amino acid production, accelerated leaf ageing leading to protein breakdown, or manipulation of nutrient transport within the plant. This study revealed the induction of numerous genes related to amino acid metabolism and its derivatives in response to infestations by both sucking and chewing insects (Figure 10B, Supplementary File S2). During aphid infestation, glutamine synthetase family protein genes, *probable aminotransferase TAT2*, *tryptophan aminotransferase-related protein 4-like*, *tryptophan synthase beta chain 1-like*, *D-amino-acid transaminase*, *pyrrolidone-carboxylate peptidase-like*, *cytidine deaminase 1-like*, *proline dehydrogenase 2*, *proline-rich receptor-like protein kinase PERK4*, *PTI1-like tyrosine-protein kinase*, *leucine-rich repeat receptor-like serine/threonine/tyrosine-protein kinase SOBIR1*, *tyrosine-protein phosphatase DSP1 isoform X1*, *protein-tyrosine-phosphatase IBR5 isoform X1*, *phenylalanine N-monooxygenase CYP79D16-lik*, and *methionine aminopeptidase 2B-like isoform X1* genes are upregulated. Whereas, in spongy moth infestation, tyrosine decarboxylase 1-like, arogenate dehydratase/prephenate dehydratase 6, and phenylalanine ammonia-lyase genes are upregulated (Figure 10B).

**Figure 11:**
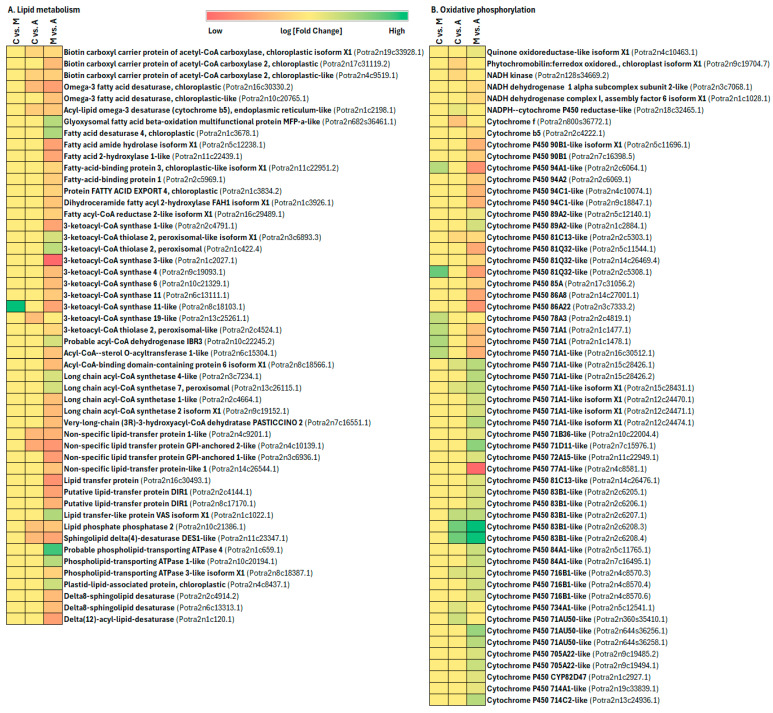
Impact of insect feeding treatment on physiologically important gene expression in Poplar leaves (treatment legend: A—aphid-infested treatment, C—control, M—moth-infested treatment). Genes are differentially regulated in (**A**) Lipid metabolism and (**B**) Oxidative phosphorylation.

Lipids are an essential class of primary metabolites that perform structural, storage, and signaling roles and serve as precursors for compounds like jasmonic acid involved in plant defense. The investigation into how maize lipids respond to feeding by Egyptian cotton worms revealed notable alterations in lipid compositions [82]. Moreover, extracts of epicuticular lipids from plants, along with specific lipid components like cutin and wax, play crucial roles in plant defense against insects by influencing oviposition, movement, and feeding behavior [83,84,85]. Lipid signalling plays a crucial role during biotic and abiotic stresses in plants [86]. An intriguing discovery from our study revealed that genes involved in lipid metabolism (*Biotin carboxyl carrier protein of acetyl-CoA carboxylase*, *Omega-3 fatty acid desaturase*, *Acyl-lipid omega-3 desaturase (cytochrome b5)*, *Fatty acid amide hydrolase isoform X1*, *Fatty-acid-binding protein 1*, *Protein FATTY ACID EXPORT 4*, *Dihydroceramide fatty acyl 2-hydroxylase FAH1 isoform X1*, *Fatty acyl-CoA reductase 2-like isoform X*, *3-ketoacyl-CoA synthase 1-like*, *Acyl-CoA-sterol O-acyltransferase 1-like*, *Long chain acyl-CoA synthetase 2 isoform X1*, *Very-long-chain (3R)-3-hydroxyacyl-CoA dehydratase PASTICCINO 2*, *Non-specific lipid-transfer protein 1-like*, *Lipid transfer protein*, *Lipid phosphate phosphatase 2*, *Sphingolipid delta(4)-desaturase DES1-like*, *Phospholipid-transporting ATPase 3-like isoform X1*, etc.) were uniformly downregulated in sucking insect attacks, suggesting a negative correlation between lipid levels and the induced plant defense against these types of insect feeding (Figure 11A, Supplementary File S2).

**Figure 12:**
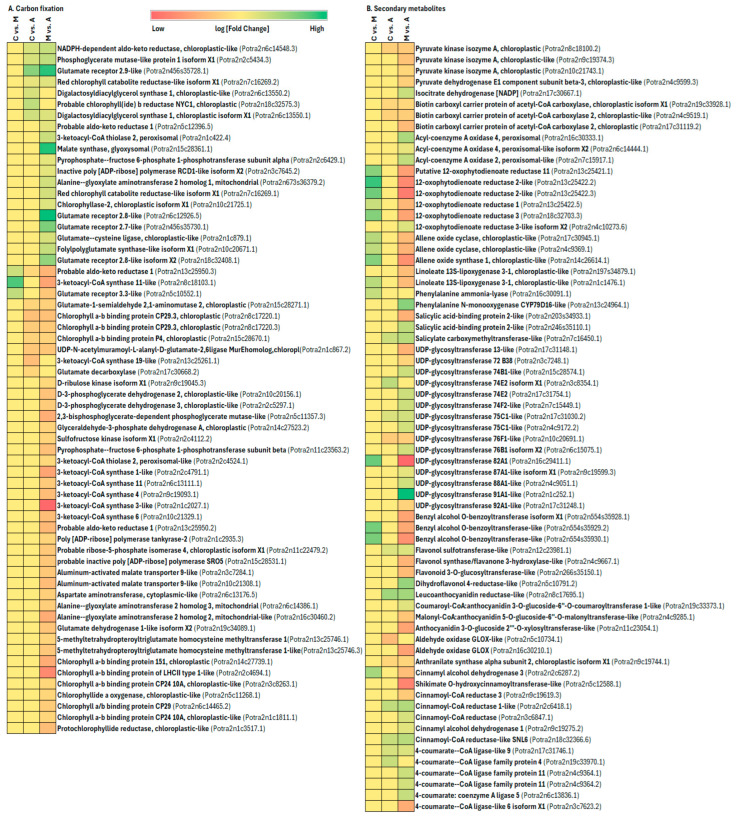
Impact of insect feeding treatment on genes related to (**A**) carbon fixation, (**B**) defense (secondary metabolite). Treatment legend: A—aphid-infested treatment, C—control, M—moth-infested treatment.

**Figure 13:**
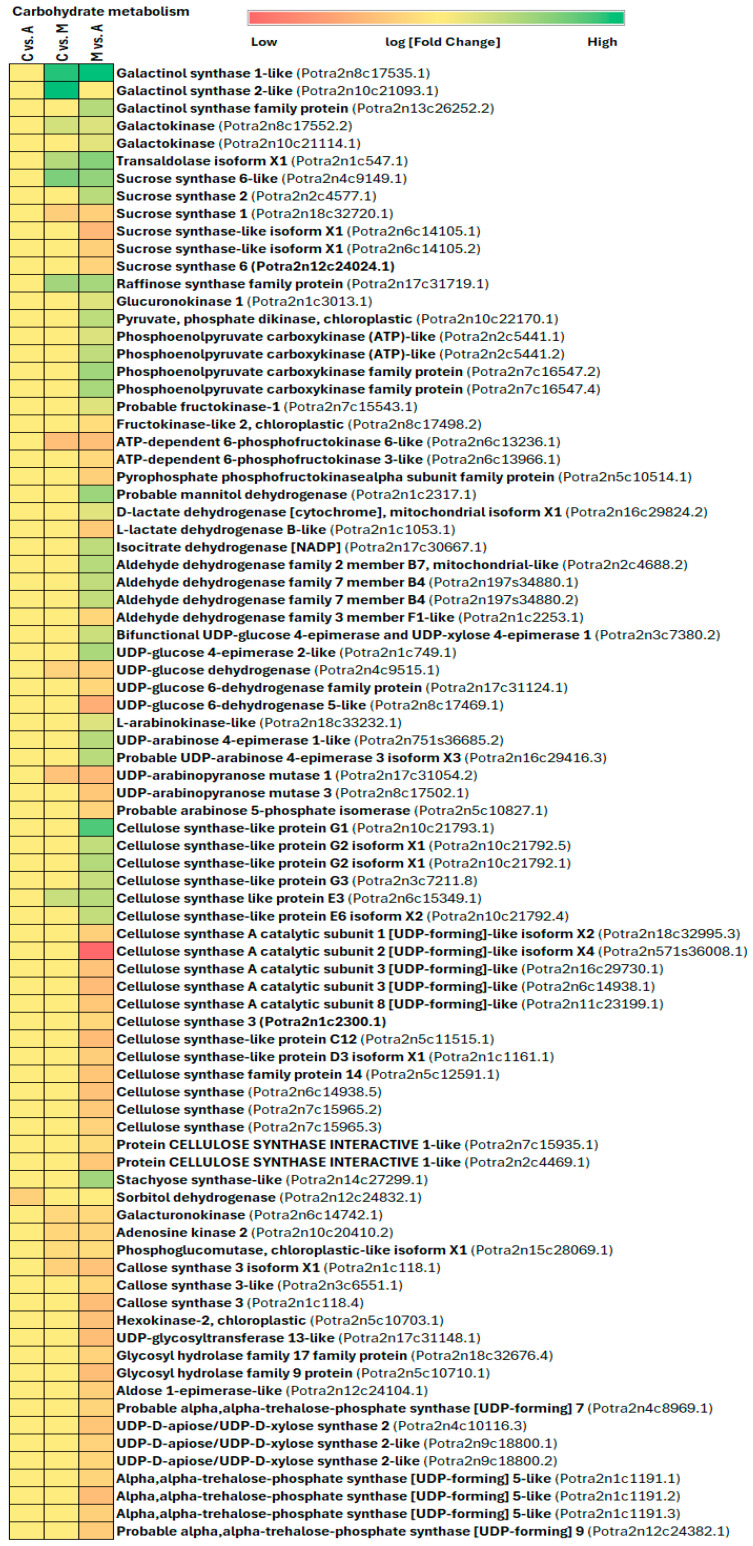
Impact of insect feeding treatment on genes related to carbohydrate metabolism and energy production under biotic stress. Treatment legend: A—aphid-infested treatment, C—control, M—moth-infested treatment.

Enhanced photosynthesis and localized carbohydrate breakdown can fuel plant defenses during interactions with herbivores [87,88]. Evidence for decreased photosynthesis due to herbivory is backed by direct measurements of alterations in photosynthesis rate, gene expression linked to photosynthesis, or the synthesis of proteins integral to the photosynthetic machinery [74,78,89]. The chewing herbivores, which consume leaf material, and phloem-feeding insects, which extract nutrients from the phloem, trigger decreased expression of genes associated with photosynthesis [87,90]. Even cues of insect presence, such as oviposition or exposure to volatile compounds emitted by infested plants, can diminish photosynthetic capacity without causing direct damage [91,92]. These findings suggest that the decline in photosynthetic activity is a deliberate response by the plant rather than merely a byproduct of metabolic constraints during herbivory. In this study, we found that many genes involved in energy metabolism (including oxidative phosphorylation and carbon fixation in photosynthetic organisms) and carbohydrate metabolism were induced by sucking and chewing insect infestation (Figure 11B, Figure 12A and Figure 13, Supplementary File S2). In aphid infestation, oxidative phosphorylation genes cytochrome P450 71A1-like, cytochrome P450 71B36-like, cytochrome P450 71D11-like, cytochrome P450 72A15-like, cytochrome P450 81C13-like, cytochrome P450 83B1-like, cytochrome P450 84A1-like, cytochrome P450 716B1-like, cytochrome P450 734A1-like, cytochrome P450 71AU50-like, cytochrome P450 705A22-like, cytochrome P450 CYP82D47, cytochrome P450 714A1-like, and cytochrome P450 714C2-like gene were upregulated, whereas, in spongy moth infestation, cytochrome P450 94A1-like, cytochrome P450 81Q32-like, cytochrome P450 78A3, and cytochrome P450 71A1 genes are upregulated (Figure 11B). In aphid infestation, carbon fixation-related genes like NADPH-dependent aldo-keto reductase, Glutamate receptor 2.9-like, Digalactosyldiacylglycerol synthase 1, Malate synthase, Pyrophosphate--fructose 6-phosphate 1-phosphotransferase subunit alpha, Chlorophyllase-2-chloroplastic isoform X1, Glutamate receptor 2.8-like, etc., were upregulated (Figure 12A, Supplementary File S2).

When herbivores attack plants, they disrupt the usual carbohydrate supply from photosynthesis, whereas to compensate, plant cells often turn to alternative carbon and energy sources to produce defensive compounds. Many plants facing herbivore threats boost the breakdown of energy storage compounds like sucrose or starch locally. For example, a study on *Arabidopsis* involving four insect herbivores found increased expression of invertases and genes responsible for breaking down complex carbohydrates [78]). Similarly, in grain amaranth (*Amaranthus cruentus*), leaf herbivory led to a rise in cytoplasmic invertase and amylolytic enzyme activities and decreased monosaccharides concentrations like sucrose and starch in the affected tissues in the days following herbivore infestation [93]. In the present study, aphid infestation, carbohydrate metabolism-related genes like galactinol synthase 1-like, galactokinase, transaldolase isoform X1, sucrose synthase 6-like, raffinose synthase family protein, glucuronokinase, pyruvate-phosphate dikinase, phosphoenolpyruvate carboxykinase family protein, D-lactate dehydrogenase, isocitrate dehydrogenase, aldehyde dehydrogenase family 2 member B7, aldehyde dehydrogenase family 7 member B4, bifunctional UDP-glucose 4-epimerase and UDP-xylose 4-epimerase, UDP-glucose 4-epimerase 2-like, cellulose synthase-like protein G1, stachyose synthase-like, etc., were upregulated (Figure 13, Supplementary File S2).

These findings underscore the significant involvement of primary metabolite pathways, such as carbohydrate, lipid, amino acid metabolism, oxidative phosphorylation, and carbon fixation, in the defense response of *P. tremula* to sucking and chewing insects.

### 3.4. Plant Secondary Metabolism Alteration Due to Aphid and Spongy Moth Infestation

**Figure 14:**
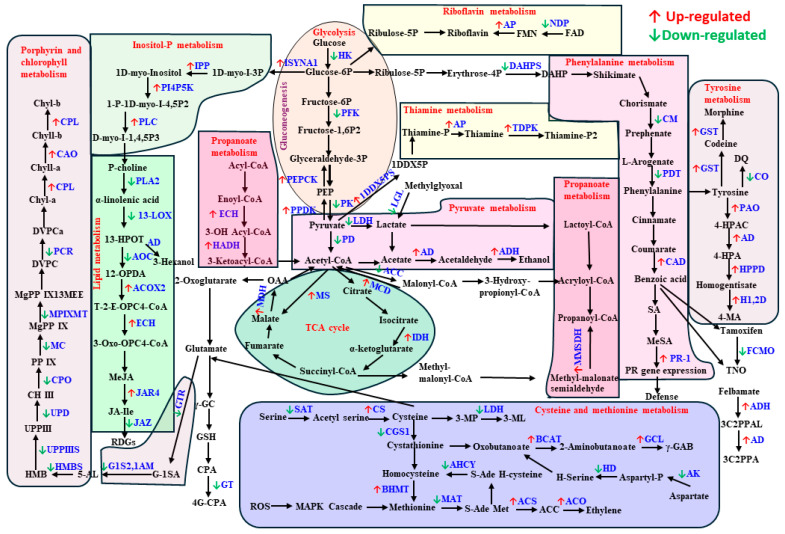
Diagrammatic representation of DEGs analysis in the aphid-infected leaf samples compared to spongy moth-infected leaf samples. The red (↑) and green (↓) arrows represent the up- and down-regulated genes (*p*-value < 0.05) in aphid-infested leaf samples. The DEGs coding enzymes are mainly related to carbohydrate metabolism (glycolysis-gluconeogenesis, pyruvate metabolism, citrate cycle, propanoate metabolism, inositol phosphate metabolism) lipid metabolism (alpha-linolenic acid), amino acid metabolism (phenylalanine, tyrosine, and tryptophan biosynthesis, cysteine and methionine metabolism, tyrosine metabolism), metabolism of cofactors and vitamins, other secondary metabolites synthesis (isoquinoline alkaloid biosynthesis) and xenobiotics biodegradation and metabolism (drug metabolism-cytochrome p450). Abbreviations: HK—Hexokinase; PFK—6-phosphofructokinase; PK—Pyruvate kinase; PPDK—Pyruvate, phosphate dikinase; PD—Pyruvate dehydrogenase; AD—Aldehyde dehydrogenase; ADH—Alcohol dehydrogenase; LDH—L-lactate dehydrogenase; PEPCK—Phosphoenolpyruvate carboxykinase; LGL—Lactoylglutathione lyase; ACC—Acetyl-CoA carboxylase; MS—Malate synthase; MDH—Malate dehydrogenase; IDH—Isocitrate dehydrogenase; MMSDH—Methylmalonate-semialdehyde dehydrogenase; MCD—Malonyl-CoA decarboxylase; HADH—3-hydroxyacyl-CoA dehydrogenase; ECH—Enoyl-CoA hydratase; ACOX2—Acyl-coenzyme A oxidase 2; IPP—Inositol-phosphate phosphatase; PLC—Phospholipase C; ISYNA1—Inositol-3-phosphate synthase 1; PI4P5K—1-phosphatidylinositol-4-phosphate 5-kinase; AOC—Allene-oxide cyclase; PLA2—Phospholipase A2; ECH—Enoyl-CoA hydratase; 13-LOX—13-lipoxygenase; JAR4—Jasmonoyl—L-amino acid synthetase JAR4; JAZ—Jasmonate-zim-domain protein 5; PDT—Prephenate dehydratase 6; DAHPS—Phospho-2-dehydro-3-deoxyheptonate aldolase 1; CM—Chorismate mutase; CAD—Cinnamyl alcohol dehydrogenase; CGS1—Cystathionine gamma-synthase 1; CS—Cysteine synthase; MAT—Methionine adenosyltransferase; ACO—1-aminocyclopropene-1-carboxylate oxidase; ACS—1-aminocyclopropane-1-carboxylate synthase; BHMT—Betaine--homocysteine S-methyltransferase; AHCY—Adenosylhomocysteinase; HD—Homoserine dehydrogenase; AK—Aspartate kinase; BCAT—Branched-chain-amino-acid transaminase; GCL—Glutamate--cysteine ligase; SAT—Serine O-acetyltransferase; LDH—L-lactate dehydrogenase; CO—Catechol oxidase; H1,2D—Homogentisate 1,2-dioxygenase; PAO—Primary-amine oxidase; HPPD—4-hydroxyphenylpyruvate dioxygenase; AP—Acid phosphatase; TDPK—Thiamine diphosphokinase; NDP—Nucleotide diphosphatase; HMBS—Hydroxymethylbilane synthase; MPIXMT—Magnesium protoporphyrin IX methyltransferase; CPO—Coproporphyrinogen oxidase; UPD—Uroporphyrinogen decarboxylase; G1S2,1AM—Glutamate-1-semialdehyde 2,1-aminomutase; UPPIIIS—Uroporphyrinogen-III synthase; GTR—Glutamyl-tRNA reductase; CAO—Chlorophyllide a oxygenase; CPL—Chlorophyllase; PCR—Protochlorophyllide reductase; MC—Magnesium chelatase; GST—Glucuronosyltransferase; FCMO—Flavin-containing monooxygenase; GT—Glutathione transferase. PR-1—Pathogenesis related protein-1. G-1SA—Glutamate-1-semialdehyde; 5-AL—5-Amino-levulinate; HMB—Hydroxymethylbilane; UPPIII—Uroporphyrinogen III; CHIII—Coproporphyrinogen III; PPIX—Protoporphyrin IX; MgPPIX—Mg-protoporphyrin IX; MgPPIX13MEE—Mg-protoporphyrin IX 13-monomethyl ester; DVPC—Divinylproto-chlorophyllide; DVPCa—Divinylproto-chlorophyllide a; Chyl-a—Chlorophyll a; Chyll-a—chlorophyllide a; Chyll-b—chlorophyllide b; Chyl-b—Chlorophyll b.

Insect pests have evolved several adaptive defense mechanisms to survive the morphological and biochemical phenomena that plants have created to endure their damage. The two most crucial plant defensive characteristics in terms of enhancing protection against insects are the plant’s nutritional content and its inducible and constitutive chemical barriers [94]. Plants have evolved special behaviors and life cycles to overcome the mechanical barrier, but because the chemical defense is so dynamic and costly, it is more challenging to adopt. Plants continually generate secondary metabolites as a defense mechanism, diminishing their vulnerability to insect herbivores or negatively influencing insect biology and behaviour [9]. Plant secondary metabolites are categorized according to their structural composition, and the varied pathways involved in their biosynthesis include terpenes, phenolics, as well as nitrogen- and sulfur-containing compounds, showcasing a diverse array of chemical defences [95]. While plant secondary metabolites, such as alkaloids, glucosinolates, or phenolic compounds, play a crucial role in defending against insects, their content and distribution vary significantly among plant genotypes [96,97,98,99]. Flavonoids are crucial as secondary metabolites in safeguarding plants against pathogens, herbivores, and ultraviolet radiation [100]. When herbivores attack tea plants, a large number of genes involved in the biosynthesis of flavonoids are activated, leading to an increase in the contents of flavonols, dihydroflavonols, flavan-3-ols, anthocyanidins, flavones, and flavonoid glucosides, including myricetin, rutin, dihydroquercetin, and dihydromyricetin. In contrast, some flavonoid precursors and derivatives are decreased [101,102]. Subsequent research revealed that an artificial diet supplied with quercetin glucoside decreased the larval growth rate and that an *Ectropis grisescens* infestation markedly enhanced the accumulation of quercetin glucosides generated from quercetin catalysed by UGT89AC1 [103]. Additionally, during *Empoasca onukii* infestation, the levels of tricetin, kaempferol 3-O-glucosylrutinoside, and methyl 6-Ogalloyl-b-D-glucose, along with the expression of key genes involved in flavonoid biosynthesis, were significantly increased [104]. The present study demonstrated that, in comparison to aphid infestation, spongy moth infestation led to a notable abundance of secondary metabolites such as catechin, 2-caffeoylisocitrate, proanthocyanidin 1, fructose phosphate, heptamethoxyflavone, proanthocyanidin 3, benzoic acid, methyl salicylate, apigenin, and O-caffeoyl-o-methylquinic acid (Table 1). The findings of our transcriptome and metabolome analysis demonstrated that the infestation of sucking and chewing insects stimulated the pathways involved in the manufacture of flavonoids and phenylpropanoids (Figure 12B). In aphid infestation, secondary metabolites related genes like acyl-coenzyme A oxidase 4, acyl-coenzyme A oxidase 2, Phenylalanine N-monooxygenase CYP79D16-like, Salicylate carboxymethyltransferase-like, UDP-glycosyltransferase 74B1-like, Flavonol sulfotransferase-like, Dihydroflavonol 4-reductase-like, Leucoanthocyanidin reductase-like, Cinnamoyl-CoA reductase, Cinnamyl alcohol dehydrogenase 1, 4-coumarate--CoA ligase family protein 4, etc., were upregulated (Figure 12B). Whereas, in spongy moth infestation, putative 12-oxophytodienoate reductase 11, 12-oxophytodienoate reductase 1, Allene oxide cyclase, Allene oxide synthase 1, Linoleate 13S-lipoxygenase 3-1, Phenylalanine ammonia-lyase, UDP-glycosyltransferase 82A1, Benzyl alcohol O-benzoyltransferase-like, and cinnamyl alcohol dehydrogenase 3 genes were up-regulated (Figure 12B). A comprehensive diagram of captured up/downregulated genes for specific metabolic pathways is shown (Figure 14).

This result is in line with recent research that found sensitive and resistant plant cultivars to spongy moths, and aphid infestations can control the expression of genes in the flavonoid biosynthesis pathways to produce the production of defence genes and proteins [105,106,107]. Comparable outcomes have been found in cotton plants with various pest infections [5,108,109]. Therefore, the activation of genes and metabolites linked to flavonoid production in *P. tremula* showed their possible role in inducing plant defence in *P. tremula* in response to both sucking and chewing insects.

## 4. Materials and Methods

### 4.1. Plants and Insects

The experiments utilized genetically uniform *Populus tremula* individuals aged eight months. The seeds were obtained by controlled crossing of parent trees [locations: Krušné hory (Fláje, 50.6653750N, 13.5753711E), Czech Republic, 40–50 years old]. The material from one seed was used for the in vitro propagation of genetically uniform individuals.

**Figure 15:**
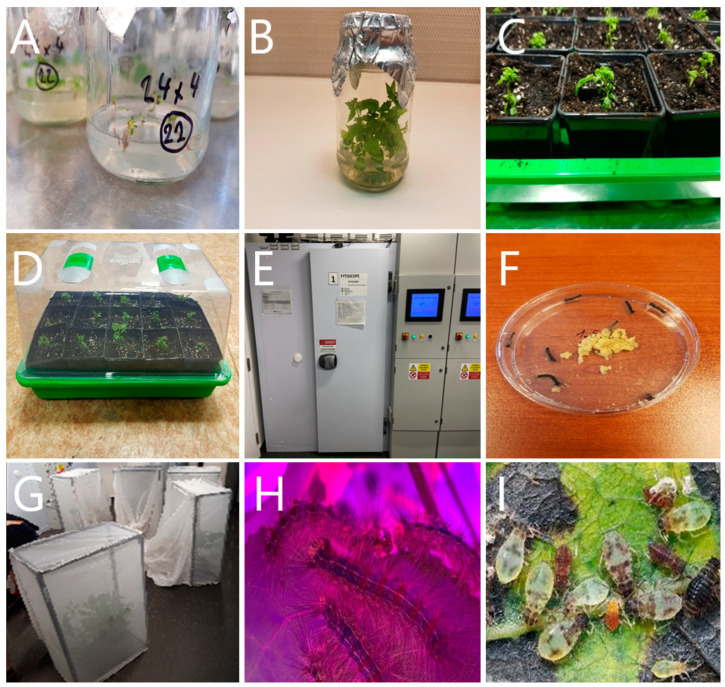
Experimental setup and plant treatment with insect feeding. (**A**) Poplar tissue propagation on MS medium. (**B**) In vitro growth of genetically uniform poplars. (**C**,**D**) Transfer of in vitro poplars to ex vitro. (**E**) Poplar plants are grown in the growth chamber. (**F**) Spongy moth larvae. (**G**) Aphid treatment setup. (**H**) Spongy moth feeding on poplar leaf. (**I**) Aphids feeding on poplar leaf.

Seeds of *P. tremula* were washed in 200 mL distilled water with 1–2 drops of Tween 20^®^ for 10–15 min, then sterilized in 0.1% HgCl_2_ for 6 min [110]. After rinsing, seeds were placed in jars with Murashige and Skoog [111] (MS) medium solidified with Danish^®^ agar and supplemented with myo-inositol and 6-benzylaminopurine (BAP). The pH-adjusted medium was autoclaved, and explants were cultivated under 16/8 h light/dark with a temperature of 22 ± 1/20 ± 1 °C (Figure 15A,B). Germination occurred within 1–3 weeks. Shoots were subcultured every 2–3 weeks until sufficient material was obtained. In vitro, rooting was done on segments with at least three buds using a half-strength MS medium supplemented with indole-3-butyric acid (IBA). Roots developed after about 4 weeks, and after 6–8 weeks, rooted shoots were transferred ex vitro (Figure 15C). Rooted shoots were washed and transferred to a sterile substrate in plastic pots, treated with Previcur Energy^®^, and cultivated under controlled conditions (Figure 15D). Humidity was gradually decreased, and plants were fertilized bi-weekly during growth.

Poplars were grown on a high-temperature steam-disinfected substrate without fungi, mold, and insect contamination (Forestina, Czech Republic) in growth chambers Step-In FytoScope FS-SI (Photon Systems Instruments, Drasov, Czech Republic) (Figure 15E). The growth chamber environment simulated optimal conditions for growth, parameters: humidity: 75%; intensity of Photosynthetic Photon Flux density: 250 µmol·m^2^·s^−1^; CO_2_ concentration: 415 ppm; day and night period: 2 h dawn, 10 h light, 2 h twilight, 10 h dark. The basic features of the experiment are the tripartite design, which includes control, leaf-chewing (*Lymantria dispar*), and phloem-feeding (*Chaitophorus populialbae*). To prevent chemical communication between different experimental plants (Poplars), 20 plants for each treatment were placed in separate growth chambers throughout the experiment. We used the spongy moth (*Lymantria dispar*, Lepidoptera: Erebidae) as a representative species of leaf-chewing insect guild. Eggs of spongy moths (*Lymantria dispar*) were supplied by the Institute of Forest Entomology, Forest Pathology, and Forest Protection at the University of Natural Resources and Life Sciences in Vienna from sterile laboratory cultures. After hatching, larvae were given a nutritionally balanced agar diet (*Lymantria dispar* agar, Southland Products Inc., Newark, DE, USA) in sterile Petri dishes (Figure 15F,H).

As a phloem-sucking insect, we used *Chaitophorus populialbae*, (Hemiptera: Aphididae), which was caught in the wild while sucking on *P. tremula* and incubated in sterile rearing containers before being placed in the growth chamber. In vitro cultures of poplar individuals served as a food source for aphids, which were replaced with fresh ones every 3 days, and at the same time, new and disinfected rearing containers were replaced (Figure 15I). This breeding method effectively reduced the risk of phytopathological contamination, especially the overgrowth of mold and fungi.

### 4.2. Experimental Design

The strategic goal of this experimental design is to ensure that the influence of insect herbivory will be the only factor that affects plant metabolism. The division into treatment groups is as follows: Control—individuals without any damage; individuals attacked by aphids (leaf-sucking); individuals attacked by a spongy moth (leaf-chewing) (Supplementary Figure S1). Before starting the experiment, five healthy (8-month-old) Poplars were selected for each group based on phenotypic characteristics. They were placed in three growth chambers separately for each group. At this stage, the poplars from the leaf-sucking treatment were placed in prepared boxes (40 cm × 100 cm × 120 cm), and the walls were made of very fine mesh (<0.01 mm), which is certified for use in the food industry (without emission of chemical substances) and does not change the spectral properties of light. The plants were left for 14 days in the climate chambers for acclimatization before the start of the experiment.

### 4.3. Poplar Tissue Feeding, Collection and Processing

The leaf-chewing treatment was formulated to capture initial occurrences of gene expression while reducing discrepancies due to leaf age and the extent of insect damage. At the same trunk level, each individual was assigned a leaf on which five spongy moth caterpillars were placed. It has been determined that approximately 30% of the leaf area must be eaten within one hour. In the test experiments, it proved critical to ensure the feeding activity of the caterpillars. They were incubated in the dark without food for 48 h to increase feeding activity, considering their nocturnal behaviour [112]. During the experiment, caterpillars were held onto the selected leaf using a size 0 goat hair brush and washed thrice in chloroform. After feeding, the leaf was cut with disinfected scissors, placed in a 50 mL falcon tube, and placed in a liquid nitrogen bath.

Treatment with aphids (*Chaitophorus populialbae*) was different due to the significantly weaker and different effect compared to caterpillars on the plant [113,114]. Part of the aphids were moved from the reared colonies using a prepared size 0 goat hairbrush (treated with chloroform and adequately ventilated). Part of the aphids were moved from the reared colonies using a prepared size 0 goat hairbrush (treated with chloroform and adequately ventilated). After 2 days, the same old, fully matured leaf was taken from each poplar. Aphids and remnants of aphid bodies were removed using a goat hairbrush—it was then immediately placed in a 50 mL falcon tube and placed in a liquid nitrogen bath.

Plant tissue samples were stored at −80 °C for further processing. After lyophilization and homogenization, the processed plant tissue was divided into two halves. One half was intended for non-targeted metabolomics analysis, and the other was used for RNA isolation and subsequent transcriptomic analysis and RT-qPCR validation.

### 4.4. Metabolomics Non-Targeted Analysis

#### 4.4.1. Extraction Procedure

Accurately 10 mg of freeze-dried and homogenized plant tissue was weighed into a 2 mL microcentrifuge tube before adding 0.5 mL of 70% cold methanol. After 30 s of vortexing, the test tube was placed into an ultrasonic bath with ice for 10 min. The solution was then centrifugated for 10 min at 13,000 rpm and 4 °C. The supernatant was filtered using a 0.22 µm PTFE filter before LC-MS-qTOF analysis. All manipulations with samples were performed on the ice.

#### 4.4.2. LC-MS-qTOF Metabolomic Analysis

Metabolomic analysis using LC-MS-qTOF was performed utilizing an Agilent 1290 Infinity II system coupled with an Agilent 6546 LC/MS QTOF instrument (Agilent, Santa Clara, CA, USA). A column of InfinityLab Poroshell 120 EC-C18 (2 × 150 mm, 2.7 µm) from Agilent (USA) was employed. The mobile phase consisted of two components: mobile phase A containing 0.1% formic acid and 0.005 M ammonium fluoride, and mobile phase B comprising acetonitrile and 0.01% formic acid. The gradient elution program consisted of the following proportions: 0–4 min, 85% A; 4–7 min, 75%; 7–9 min, 68% A; 9–16 min, 60% A; 16–22 min, 45% A; 22–28 min, 5% A; 28–30 min, 5% A. The flow rate of the mobile phase was set to 0.5 mL min^−1^, and the column temperature was maintained at 35 °C. A 1 µL injection volume was used. The system operated in both positive and negative ionization modes. The QTOF parameters were configured as follows: scan range of 100–1000 *m*/*z*; the drying gas temperature at 160 °C; sheath gas flow rate of 12.0 L/min; sheath gas temperature at 400 °C; capillary voltage set to 5.0 kV; nozzle voltage at 2.0 kV; fragmentor set to 140 V; collision energy employed at 10, 20, and 40 eV. MS/MS data were acquired with a scan range of 50–800 *m*/*z*, a retention time window of 0.5 min, an isolation window of 1.3 amu, and an acquisition rate of 3 spectra per second. For mass correction, the analysis monitored two reference masses, 112.9855 *m*/*z*, and 966.0007 *m*/*z*.

The raw data files were processed using Mass Hunter Profinder 10.0 software for time alignment and feature extraction. Parameters for time alignment were set as minimal intensity 1000 counts and maximum time shift 0.5 min plus 0.3%. For feature extraction, the parameters were *m*/*z* range 100–1000, minimal intensity 1000 counts, retention time tolerance 0.25 min, and mass tolerance 20 ppm plus 2 mDa.

The obtained data were exported to Metabolanalyst (https://www.metaboanalyst.ca/ (accessed on 13 November 2021)) for statistical analysis and visualization. The data were filtered by interquartile range, normalized by a median, log-transformed, and mean centering on identifying metabolite target MS/MS analyses. Metabolite identification was performed by comparing data from the Metline Database, internal library, and literature according to the retention time and MS/MS fragmentation.

#### 4.4.3. Statistical Evaluation of LC-MS-qTOF Data

Separated signals were aligned, and data from three injections of each sample were averaged. Constant sum normalization was performed, followed by the centred log-ratio (clr) transformation. Principal Component Analysis (PCA) and Orthogonal Partial Least Square Discriminant Analysis (OPLS-DA) of Pareto-scaled data were created in Simca 17.0 SW (Sartorius Stedim Data Analytics AB, Sweden). To select the most affected metabolites, separate OPLS-DA models were constructed for control vs. moth-infested, moth vs. aphid-infested, and control vs. aphid-infested leaves. From the variable importance for projection (VIP) plot, compounds with a VIP value higher than 1, at least in one of the two-group models, were selected for their metabolism pathways evaluation.

### 4.5. Transcriptomics Analysis

#### 4.5.1. Total RNA Isolation

For the RNA isolation, leaf samples (50 mg) were put into 2 mL Eppendorf Safe-Lock tubes containing three steel grinding balls and frozen under liquid nitrogen. Subsequently, the tissue was ground with Retsch Mixer Mill 400. Total RNA was extracted with Epicentre MasterPure RNA Purification Kit (Epicentre). After extraction, the total RNA underwent DNase I treatment using the TURBO DNase Kit (Ambion, Austin, TX, USA). Subsequently, the integrity of the purified total RNA was assessed on a 1.2% agarose gel, and its concentration was determined using the NanoDrop spectrophotometer (Thermo-Fisher Scientific, Waltham, MA USA).

#### 4.5.2. NGS Sequencing and Data Analysis

Transcriptome libraries were constructed using leaf samples infected with aphids, spongy moths, and respective control leaf samples (Supplementary Figure S1). To enrich mRNA, oligo (dT) beads were employed, followed by cDNA library preparation using the NEB Next^®^ Ultra™ RNA Library Prep Kit and Illumina Novaseq6000 sequencing, resulting in 30 million reads (150 paired ends) per sample. Each sample had five biological replicates. Differential gene expression analysis (DGE) was conducted by mapping raw reads to the *P. tremula* reference genome [27] using the OmicsBox transcriptomics module (ver 1.4.11) following the developer protocol as described thoroughly in our latest publication [115]. DGE was performed using pairwise differential expression analysis in OmicsBox, which was based on edgeR software package (Bioconductor project) [116], deploying a negative binomial Generalized Linear Model (GLM) for multi-factorial statistical analysis to identify differentially abundant transcripts, with FDR corrected *p*-value < 0.05 and fold change ±2 as thresholds for differentially expressed transcripts (DETs). To illustrate the expression pattern using Cluster 3.0, hierarchical clustering was performed using the average linkage approach with Euclidean distance based on log fold change data [117].

#### 4.5.3. Reference Gene Selection for RT-qPCR

To identify the optimal reference gene for gene expression validation and perform RT-qPCR studies, preliminary studies were conducted, considering genes previously reported and commonly utilized in *P. tremula*. Seven genes were chosen from the transcriptomic data of *P. tremula*, comprising *polyubiquitin* (Ubiquitin), *glyceraldehyde-3-phosphate dehydrogenase* (GAPDH), *Actin 7* (Act7), *serine*/*threonine-protein phosphatase 2A* (PP2A), *elongation factor 1-beta 1* (EF1B1), *elongation factor 1-alpha* (EF1A), and *tubulin beta-4 chain-like* (Tubulin 4) (Supplementary Table S1). The sequences retrieved underwent a BLASTx search against the NCBI database to corroborate their annotations. One microgram of total RNA was utilized for cDNA synthesis employing the High-Capacity cDNA Reverse Transcription kit (Applied Biosystems-Life Technologies, Waltham, MA, USA), stored at −20 °C. Before usage as a template in RT-qPCR experiments, the cDNA samples underwent a 10-fold dilution. Each RT-qPCR assay involved four biological replicates per sample. Primer design was conducted using the IDT PrimerQuest software (IDT, Belgium, https://sg.idtdna.com/pages/tools/primerquest?returnurl=%2FPrimerquest%2FHome%2FIndex (accessed on 13 November 2021)) (Supplementary Table S1). RT-qPCR analyses were conducted for all samples, including controls and treatments. The 10 μL RT-qPCR reactions comprised 5.0 μL SYBR^®^ Green PCR Master Mix (Applied Biosystems), 1.0 μL cDNA, 1.0 μL of 10 μM forward and reverse primers, and 3.0 μL RNase-free water (Invitrogen, Waltham, MA, USA). Reactions were conducted in an Applied Biosystems™ StepOne™ Real-Time PCR System (Applied Biosystems) under the following conditions: initial denaturation at 95 °C for 10 min, followed by 40 cycles of 95 °C for 15 s, 60 °C for 1 min, and dissociation curve analysis with temperature increasing from 60 to 95 °C. Target gene expression levels were determined using the 2^–ΔΔCt^ method [118].

The selection of the most effective reference gene was based on assessing their expression stability using standard algorithms described by earlier studies [119]. Utilizing algorithms such as geNorm, Normfinder, Bestkeeper, Delta CT, and RefFinder, the stability of gene expression was assessed to identify the most suitable reference genes for precise normalization of target gene expression across leaf samples affected by aphids and spongy moths and control samples.

#### 4.5.4. Gene Expression Validation by RT-qPCR

To verify the expression of target genes across treatment and control samples, we selected twenty genes linked to both up- and downregulation such as *Endochitinase EP3* (ECEP3), *Glucan endo-1,3-beta-glucosidase, basic isoform* (G1,3BGLU), *Symbiosis receptor* (SRLK), *Vacuolar-sorting receptor 6* (VSR6), *Expansin-like B1* (EB1), *Gibberellin-Insensitive Dwarf1* (GID1b), *Auxin response factor 5.2* (ARF5), *Transcription factor MYB59-like isoform X2* (MYB59), *Probable inorganic phosphate transporter 1* (IPT), *Pathogenesis-related protein* (PRP), *Caffeoylshikimate esterase* (CE), *NAC domain-containing protein 21*/*22-like isoform X2* (NAC21), *B-box zinc finger protein 32* (BZFP32), *Proline dehydrogenase 2, mitochondrial* (PD2), *Transcription factor bHLH137-like isoform X1* (bHLH137), *Galactinol synthase 1* (GS1), *Cytochrome P450 83B1* (CYP450-83B1), *Probable carboxylesterase 8* (PC8), *Protein P21* (PP21) and *Probable nucleoredoxin 2 isoform X2* (NR2) in the transcriptomic data (Supplementary Table S2). The RT-qPCR study was performed using four biological replicates from each treatment using the same protocol described before. The RT-qPCR expression data were normalized using the PP2A reference gene. A one-way ANOVA test was performed to evaluate the significance of gene expression differences in RT-qPCR.

## 5. Conclusions

**Figure 16:**
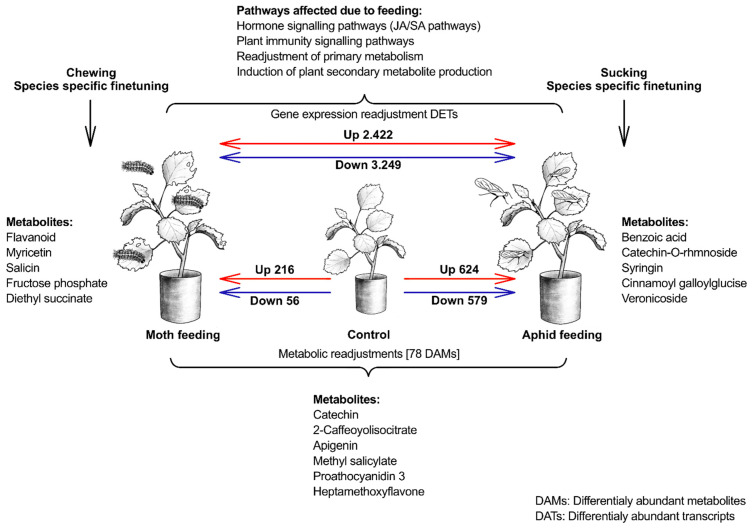
Summary of the poplar defence against two different insects (i.e., spongy moth and aphid) obtained from current metabolomic and transcriptomics study. Our finding indicates that distinct metabolic pathways and gene expression from key physiological pathways in poplar leaves are altered after insect attack, suggesting a species-specific, fine-tuned response.

Sucking and chewing insects feeding on *P. tremula* trigger notable changes in the *P. tremula* physiology. Through an integrated analysis of both transcriptome and metabolome (Figure 16), it was observed that pathways related to flavonoid and isoflavonoid biosynthesis are significantly enriched in response to sucking and chewing insect infestation.

Moreover, crucial pathways like plant hormone signal transduction (salicylic acid and jasmonic acid), PAMP-triggered immunity, and MAPK signalling pathway–plant interactions play pivotal roles in inducing plant resistance against both sucking and chewing insects in *P. tremula*. Additionally, insect infestation prompts various alterations in plant primary metabolism, particularly in carbohydrate and amino acid pathways, compared to non-infested plants. These findings enhance our current understanding of how plants respond to herbivore-induced stress and offer insights for developing strategies to combat aphids and spongy moths in *P. tremula*.

## Figures and Tables

**Table 1:** Top 10 metabolites differentially abundant between treatments.

Control Leaves vs. Moth-Infested Leaves			Control Leaves vs. Aphid-Infested Leaves			Aphid-Infested Leaves vs. Moth-Infested Leaves		
Metabolite	VIP Value	KO Number	Metabolite	VIP Value	KO Number	Metabolite	VIP Value	KO Number
Myricetin 3-2	1.7	00941	Trihydroxypentamethoxyflavone	2.2	00941	Catechin	1.8	00941
Trihydroxypentamethoxyflavone	1.6	00941	Benzoic acid	2.2	00362	2-Caffeoylisocitrate	1.7	00940
Diethyl succinate	1.6	00020	Cinnamoyl galloylglucose	2.1	00941	Proanthocyanidin 1	1.6	00942
Cinnamoyl galloylglucose	1.6	00941	Quercetin-O-glucuronide derivate	2.1	00941	Fructose phosphate	1.6	00010
Flavonoid 5	1.5	00941	Catechin-O-rhmnoside	1.8	00941	Heptamethoxyflavone	1.5	00941
Salicin	1.4	00940	Syringin	1.7	00940	Proanthocyanidin 3	1.5	00942
Quercetin 3,5-digalactoside	1.4	00941	Veronicoside	1.6	00941	Benzoic acid	1.4	00362
Fructose phosphate	1.4	00010	Butyl O-caffeoylquinate	1.5	00940	Methyl salicylate	1.3	00940
Di-O-caffeoylquinic acid	1.4	00940	O-Vanilloylvitexin	1.5	00940	Apigenin	1.3	00941
Quercetin-O-glucuronide derivate	1.4	00941	Isorientin 3′,6′di-O-glucoside	1.5	00941	O-Caffeoyl-O-methylquinic acid	1.3	00940

KO Numbers: ko00941—Thiamine metabolism; ko00020—Citrate cycle (TCA cycle); ko00940—Phenylpropanoid biosynthesis; ko00010—Glycolysis/Gluconeogenesis; ko00362—Benzoate degradation; ko00942—Anthocyanin biosynthesis.

**Table 2:** The candidate housekeeping genes are ranked according to their stability value by geNorm, NormFinder, BestKeeper, ∆CT, and RefFinder analysis. M—the gene expression stability measure; SD—standard deviation value; SV—stability value; GM—Geomean value; and R—Ranking.

Gene Name	geNorm		NormFinder		BestKeeper		ΔCT		Comprehensive	
	M	R	SV	R	SD	R	SD	R	GM	R
Ubiquitin	1.32	3	1.334	5	2.95	6	2.14	4	4.68	5
GAPDH	0.841	1	1.002	3	2	2	2.08	3	2.06	2
Act7	1.078	2	0.785	2	2.48	5	1.9	2	2.78	3
PP2A	0.841	1	0.42	1	1.95	1	1.82	1	1	1
EF1B1	1.662	5	2.079	6	3.31	7	2.57	6	6.24	7
EF1A	2.468	6	4.331	7	2.44	4	4.48	7	6.09	6
Tub4	1.47	4	1.29	4	2.2	3	2.28	5	4.16	4

## Data Availability

The original contributions presented in the study are included in the article; further inquiries can be directed to the corresponding author. The RNA-seq raw reads were submitted under the bioproject PRJNA1084789.

## Supplementary Materials

The following supporting information can be downloaded at: https://www.mdpi.com/article/10.3390/ijms25116124/s1.
