# Supplementary material for: Genome-Wide Transcriptomic and Metabolomic Analyses Unveiling the Defence Mechanisms of Populus tremula against Sucking and Chewing Insect Herbivores
Source: Int J Mol Sci. 2024 Jun 1;25(11):6124. doi: 10.3390/ijms25116124 (PMC11172939; doi:10.3390/ijms25116124)
Supplement: Supplementary file 1 [file ijms-25-06124-s001.zip › Supplementary Figure S1.pptx]

## Slide 1
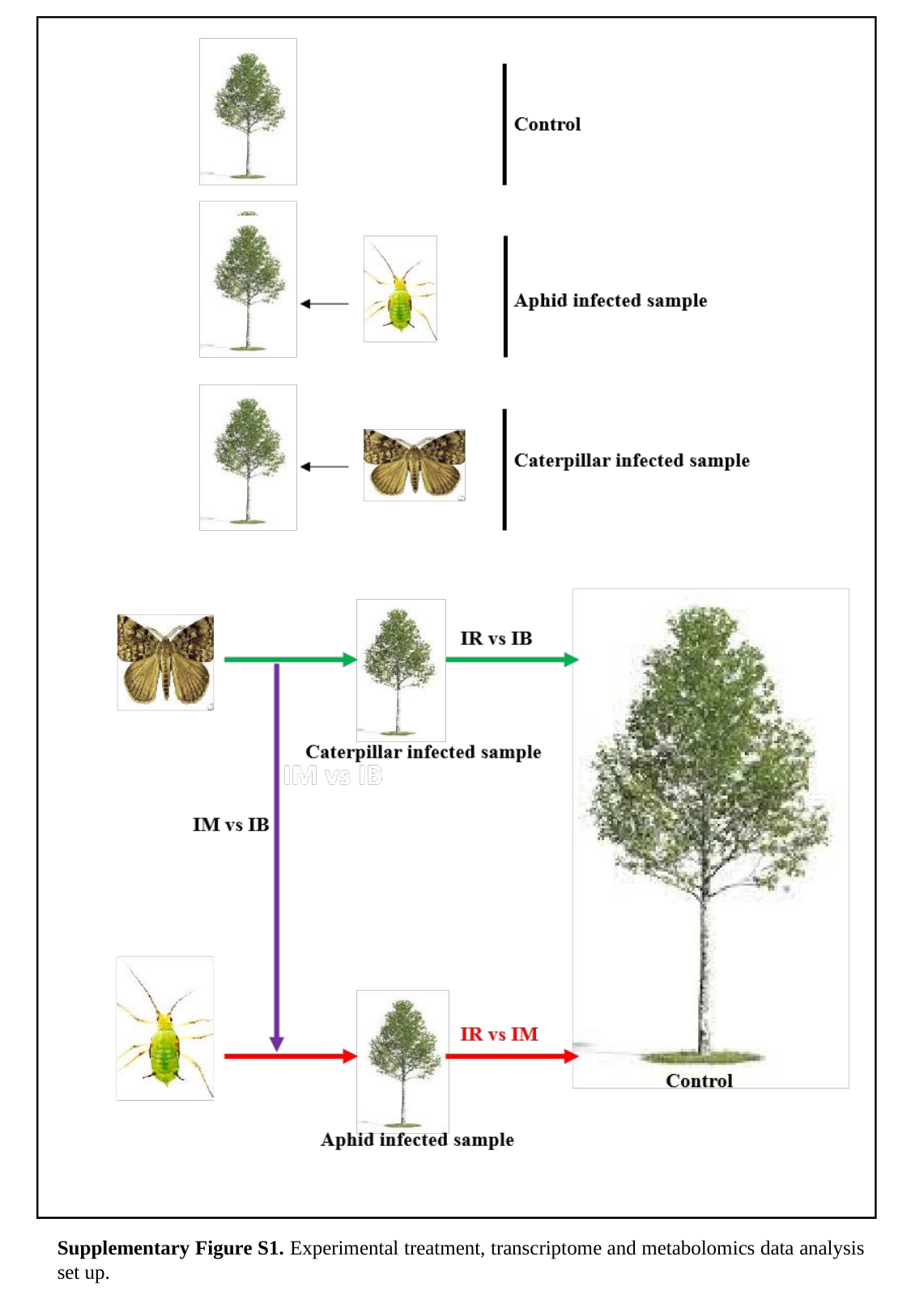

Supplementary Figure S1. Experimental treatment, transcriptome and metabolomics data analysis set up.
