# Supplementary material for: Genome-Wide Transcriptomic and Metabolomic Analyses Unveiling the Defence Mechanisms of Populus tremula against Sucking and Chewing Insect Herbivores
Source: Int J Mol Sci. 2024 Jun 1;25(11):6124. doi: 10.3390/ijms25116124 (PMC11172939; doi:10.3390/ijms25116124)
Supplement: Supplementary file 1 [file ijms-25-06124-s001.zip › Supplementary Figure S3.pptx]

## Slide 1
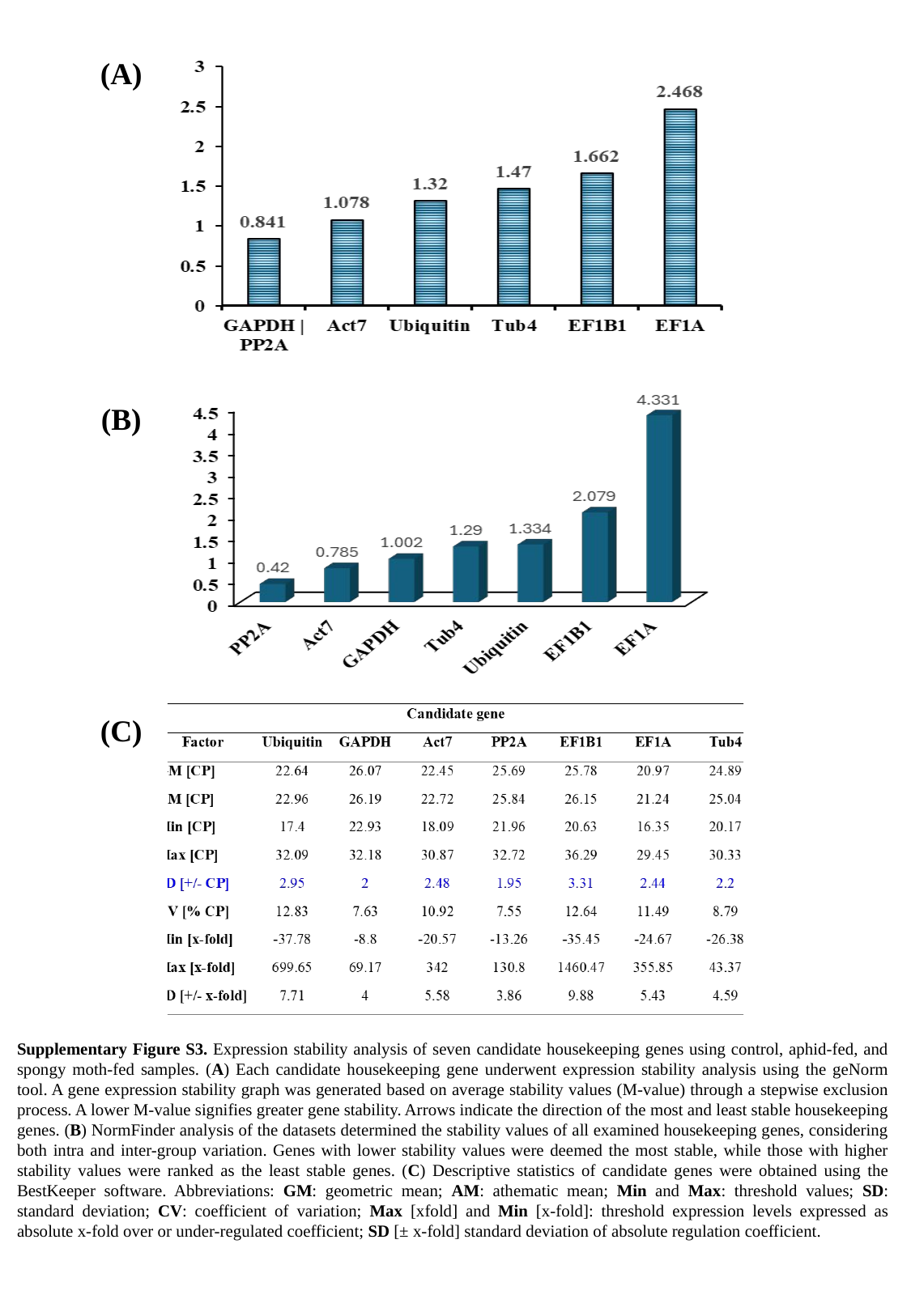

(A)
(B)
(C)
Supplementary Figure S3. Expression stability analysis of seven candidate housekeeping genes using control, aphid-fed, and spongy moth-fed samples. (A) Each candidate housekeeping gene underwent expression stability analysis using the geNorm tool. A gene expression stability graph was generated based on average stability values (M-value) through a stepwise exclusion process. A lower M-value signifies greater gene stability. Arrows indicate the direction of the most and least stable housekeeping genes. (B) NormFinder analysis of the datasets determined the stability values of all examined housekeeping genes, considering both intra and inter-group variation. Genes with lower stability values were deemed the most stable, while those with higher stability values were ranked as the least stable genes. (C) Descriptive statistics of candidate genes were obtained using the BestKeeper software. Abbreviations: GM: geometric mean; AM: athematic mean; Min and Max: threshold values; SD: standard deviation; CV: coefficient of variation; Max [xfold] and Min [x-fold]: threshold expression levels expressed as absolute x-fold over or under-regulated coefficient; SD [± x-fold] standard deviation of absolute regulation coefficient.
